# Tail of defence: an almost complete tail skeleton of *Plateosaurus* (Sauropodomorpha, Late Triassic) reveals possible defence strategies

**DOI:** 10.1098/rsos.250325

**Published:** 2025-05-21

**Authors:** Thomas Filek, Matthias Kranner, Ben Pabst, Ursula B. Göhlich

**Affiliations:** ^1^ Department of Paleontology, University of Vienna, Vienna, Austria; ^2^ Institute of Zoology, BOKU University, Vienna, Austria; ^3^ Abt.10 geologischer Dienst, Ref.102 Landesaufnahme Geologie und Geogefahren, Bayerisches Landesamt für Umwelt, Hof/Saale, Germany; ^4^ University of Zurich, Zurich, Switzerland; ^5^ Department of Geology and Paleontology, Natural History Museum Vienna, Vienna, Austria

**Keywords:** *Plateosaurus*, behaviour, defence strategy, Triassic, Frick, Switzerland

## Abstract

In 2015, a partial skeleton of the Late Triassic dinosaur *Plateosaurus trossingensis* was excavated from Frick, Switzerland, and subsequently mounted at the Natural History Museum of Vienna in 2021. This specimen includes an almost complete series of tail vertebrae, with a well-preserved, articulated whip-like distal end. The preserved tail structure provides valuable insights into the morphological implications of tail function and its potential role in the behaviour of *Plateosaurus*. Using the caudal vertebrae, we reconstructed and analysed the potential tail-lashing capabilities of *Plateosaurus*, comparing its biomechanics with those of other fossil and extant long-tailed reptilian taxa, including the extinct sauropod *Diplodocus*, the extant Asian water monitor (*Varanus salvator*), and the green iguana (*Iguana iguana*). Our results indicate that the tail of *P. trossingensis* was highly flexible, with an estimated kinetic energy output ranging between 0.537 and 1.616 kJ during rapid strikes, comparable to the defensive tail use observed in modern reptiles. These findings suggest that tail-whipping may have played a role in predator deterrence and intraspecific interactions in *Plateosaurus*.

## 1. Introduction

The Late Triassic dinosaur *Plateosaurus trossingensis* Fraas, 1912 is a plesiomorphic sauropodomorph and one of the most frequently occurring dinosaurs in Europe. Numerous partial or complete skeletons from various localities have been extensively studied [1,2]. The most fossil-rich sites include Trossingen and Halberstadt in Germany, as well as Frick in Switzerland, all of Norian age [1,3]. *Plateosaurus* has been the subject of multiple studies addressing its phylogeny, morphology, ontogeny, bone histology, sexual dimorphism, body mass, posture and range of motion [1,4–10].

These three key localities have yielded a substantial number of *Plateosaurus* specimens, with excavations uncovering approximately 80 near-complete skeletons in Trossingen, between 39 and 50 individuals in Halberstadt, and over 40 skeletons in Frick. All *Plateosaurus* specimens from Frick are currently attributed to a single species, which was historically referred to as *Plateosaurus engelhardti* [11], but was synonymized with *P. trossingensis* [12] in a taxonomic revision by Lallensack *et al*. [2].

Various authors have interpreted the accumulations of *Plateosaurus* remains as evidence of a social, herd-forming system [4,13,14]. Holtz [15] proposed that herd behaviour in herbivorous sauropods may have functioned as a defence strategy against potential predators. However, Sander [3] remains more equivocal, arguing that the accumulations of *Plateosaurus* do not necessarily confirm herd behaviour. Instead, he suggested that recurring water holes may have acted as natural traps, leading to mass mortalities. Additionally, the search for essential resources such as water, food or mates has been considered a contributing factor to the clustering of *Plateosaurus* fossils in these localities. Similar behaviour is observed in solitary-living reptiles, such as *Varanus* Merrem, 1820 [16], where individuals congregate in response to competition for resources [17].

Beyond the abundance of *Plateosaurus* fossils, isolated remains of contemporary predatory dinosaurs have been discovered at all three sites, including teeth of *Liliensternus liliensterni* Welles, 1984 [18] and partial skeletons of *Notatesseraeraptor frickensis* Zahner & Brinkmann, 2019 [1,3,19]. When comparing body size and weight, *Plateosaurus* ranged from 4.8 to 10 m in length and weighed 600 to 1000 kg [20], while potential predators such as *Liliensternus* measured approximately 5 m and weighed around 130 kg [21,22]. *Notatesseraeraptor* reached up to 3 m, though its weight remains unknown [19]. This suggests a possible predator–prey relationship, though it remains unclear whether *Plateosaurus* was actively hunted, primarily scavenged or the association is an effect of environmental association (i.e. water hole use), or taphonomy (e.g. [23]).

Given its herbivorous diet and the absence of armour or obvious defensive structures such as osteoderms, horn shields or spikes [4], *Plateosaurus* may have been comparatively more vulnerable to predation than contemporaneous taxa equipped with such features.

However, the absence of armour or other passive defences does not preclude the possibility of alternative strategies, such as herd formation or active self-defence using both the claws and tail. Given that *Plateosaurus* possessed long, curved hand claws typically associated with grasping [8], it is plausible these could have also served a defensive function (compare [24]), as seen in modern reptiles that use their forelimbs in agonistic interactions (compare [25]). In addition, the flexible and elongated tail may have been used for striking motions, adding another active mechanism for deterring predators.

At Frick, as well as at the German localities, *Plateosaurus* skeletal remains are distributed across multiple stratigraphic layers, challenging the hypothesis of herd behaviour [3,26]. Nevertheless, apart from possible social structures, *Plateosaurus* may have employed active defence mechanisms, such as tail-whipping, not only for self-protection but potentially also in parental care scenarios. The tail of *Plateosaurus* is estimated to have consisted of approximately 45 caudal vertebrae [4], accounting for ~45% of the total body length, and is thought to have been held horizontally [1]. Mallison [9] described the tail as highly flexible due to its numerous elements, allowing a lateral deflection of up to 45° in the proximal ten caudal vertebrae relative to the horizontal axis.

The use of the tail as a defensive organ has been proposed for other Triassic and Jurassic dinosaurs lacking visible protective structures (e.g. [27]). One notable example is *Diplodocus* Marsh [28], which had an exceptionally elongated tail composed of approximately 80 caudal vertebrae [27], nearly twice the number found in *Plateosaurus* [4,27]. The tail of *Diplodocus* has been hypothesized to serve a defensive function, potentially even producing supersonic cracking sounds to deter predators [29,30]. In addition, some sauropods developed specialized features such as coossified or inflated distal caudal vertebrae, which may represent structural adaptations for tail-based defence [31].

**Figure 1.**
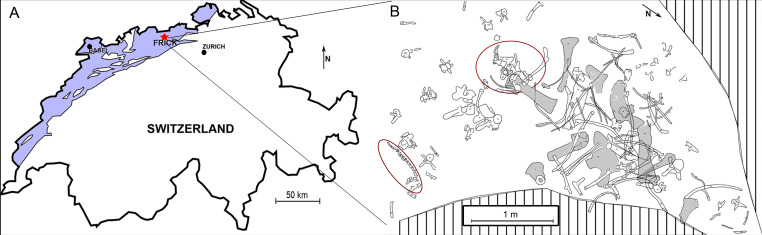
(A) Position of the Frick locality (blue area marks Jurassic carbonate which overlies Triassic strata). (B) Excavation map of all bone findings belonging to the Vienna specimen of *Plateosaurus* (Frick Field no. 15.5), now housed in the NHMW (Vienna). Red circles mark articulated bone findings such as the tail tip (34 cm long) and hindfoot.

The Vienna specimen of *Plateosaurus* (Frick Field number 15.5), excavated in Switzerland in 2015 (figure 1A,B), comprises an almost complete tail skeleton, including an exceptionally rare articulated tail tip. This well-preserved tail structure presents a unique opportunity to investigate the hypothesis of tail-whipping as an active defence mechanism in *Plateosaurus*. To test this, the lateral striking energy of the tail is calculated and compared with that of another long-tailed fossil dinosaur (*Diplodocus*) and extant reptilian taxa known to utilize their tails for defence, including *Varanus salvator* [32] and *Iguana iguana* [33], as modern reptiles exhibit a wide range of agonistic behaviours, including biting, wrestling, and tail-lashing in both territorial and defensive contexts [34]. This comparative approach enhances the understanding of potential defensive behaviours in *Plateosaurus* and contributes to the broader study of dinosaur biomechanics and behaviour [35,36].

## 2. Methods and material

### 2.1. Locality and geological background

The Frick locality is situated in northern Switzerland, in the canton of Aargau (figure 1A). Over the past 60 years, numerous partial and a few complete skeletons of *P. trossingensis* have been excavated from the Gruhalde clay pit, operated by Tonwerke Keller AG [26]. The specific location of the partial skeleton studied in this work (Frick Field no. 15.5) is recorded at the coordinates 2° 642′ 960″/1° 261′ 963″.

All *Plateosaurus* remains from Frick originate from the Upper Variegated Marls (Norian, Late Triassic), a stratigraphic unit approximately 12 m thick [2,37]. Within this section, dinosaur fossils are primarily found in three horizons: the lower, middle and upper saurian layers [2,38]. The *Plateosaurus* specimen analysed in this study (Frick Field no. 15.5) was recovered from the lower saurian layer bonebed, at a depth of approximately 2.8 m and positioned ~10 m below the boundary to the overlying Jurassic ‘Insect Marl Layer’ of the Klettgau Formation (latest Norian) (figure 1B).

### 2.2. Compared material

#### 2.2.1. The Vienna specimen of *Plateosaurus trossingensis* (Frick Field no. 15.5)

**Figure 2.**
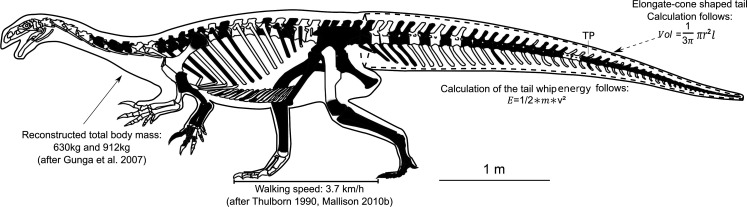
Identified bones of Vienna specimen of *P. trossingensis* (Frick Field no. 15.5) marked in black. Black outline represents the total body mass reconstructed by Gunga *et al*. [20]. Dashed line represents the calculated elongate-cone-shaped tail. TP stands for transition point, where the caudal vertebrae length exceeds their total height.

The *Plateosaurus* specimen examined in this study was unearthed in 2015 and is permanently housed at the Natural History Museum Vienna (NHMW; unnumbered, therefore the field number is used), where it was prepared, mounted and placed on public display in 2021. This partial skeleton comprises 188 postcranial bones from a single individual (figure 2) but lacks a skull. During preparation, a theropod tooth was unexpectedly discovered attached to the *Plateosaurus* pubis (element number 15.5.40/41), embedded within the sediment matrix. Among the preserved skeletal elements, two features are particularly noteworthy: (i) an articulated foot and (ii) an articulated tail tip, which is exceptionally rare among previously recovered *Plateosaurus* specimens. A total of 43 caudal vertebrae (Ca) were identified; however, several remain incomplete, with damaged neural spines and transverse processes. The total measured tail length (sum of all Ca) is 196 cm, with a whip-like tail tip measuring 60 cm beginning between Ca23 and Ca25 where vertebral length surpasses total height. In the Vienna specimen, the last 18 small caudal vertebrae are preserved in articulation. Due to the extreme rarity of well-preserved *Plateosaurus* tail endings, the original articulated tail tip is permanently stored at the Museum Frick (MF), Switzerland, while a cast of the tail tip was incorporated into the mounted skeleton at the NHMW.

#### 2.2.2. Comparative material

To assess tail whipping as a possible defensive behaviour, comparative measurements were conducted using fossil and extant reptilian taxa. The energy calculations of the *Plateosaurus* tail (table 1) were compared with those of another *Plateosaurus* specimen (after [4]), the fossil sauropod *Diplodocus carnegii* [39], the extant Asian water monitor (*V. salvator*) and the green iguana (*I. iguana*) (table 2). The latter two extant taxa are known for their tail-whipping defensive strategies [25,40,41], which has also been hypothesized for *Diplodocus* [30].

**Table 1.** Calculated energy of tail whipping for *Plateosaurus trossingensis* (specimen Frick Field no. 15.5, housed in NHMW).

	only bones	additionally added missing caudal bones	with estimated soft tissue	additionally added missing caudal bones and estimated soft tissue
tail mass (kg)	26.5	28.4	76.5	82.0
tail length (m)	1.958	2.1	1.958	2.1
mass of whiplash (from Ca25) (kg)	0.3	0.3	0.7	0.8
length from whiplash (from Ca25) (m)	0.597	0.622	0.597	0.622
impact energy for whole tail when held horizontally (kJ)	56.136	60.207	162.234	173.999
impact energy transferred from whiplash (kJ)	47.716	51.176	137.899	147.899
impact energy transferred by a quick whiplash (kJ)	0.537	0.559	1.553	1.616

**Table 2.** Measurements of caudal vertebrae and possible tail whip energy of analysed specimens.

taxa	number of caudal vertebrae	total tail length (m)	tail whip length (m)	percentage of tail whip on total tail length (%)	energy of total tail whip (only bones in kJ)
*Varanus salvator* (IPUW 3480)	110.00	1.03	0.34	32.80	0.03
*Diplocodus carnegii* (NHMW 1904/0004/0003)	70.00	13.26	7.28	54.90	179.54
*Plateosaurus trossingensis* [4]	42.00	2.55	0.48	18.70	82.51
***Plateosaurus trossingensis* (Frick Field no. 15.5)**	**42.00**	**1.90**	**0.60**	**31.50**	**56.14**
*Iguana iguana* (NHMW-ZOO-HS-1680)	39.00	0.64	0.41	37.10	0.01

##### 2.2.2.1. Plateosaurus trossingensis

The *Plateosaurus* specimen described by Huene [4] represents one of the earliest and most comprehensive osteological records of the genus. This individual, excavated from the Upper Triassic strata of the Trossingen Formation in Germany, includes a nearly complete tail composed of approximately 45 caudal vertebrae. Huene reconstructed the tail morphology with a distinctive tapering shape and noted the absence of pronounced processes in the distal caudals, consistent with a whip-like form. In his reconstruction, the whip-like portion was estimated to comprise around 40% of the total tail length. This proportion exceeds that of the Vienna *Plateosaurus* specimen, which preserves a whip-like segment measuring ~31.5% of the tail. These differences, although potentially influenced by taphonomic and reconstruction-related factors, provide a valuable basis for intra-generic comparison of caudal morphology and potential functional interpretations.

##### 2.2.2.2. Diplodocus carnegii

The mounted skeleton cast of *D. carnegii* (NHMW-Geo-1909/0004/0003, original at Pittsburgh Carnegie Museum) was reconstructed using two partial skeletons from Sheep Creek, Wyoming (discovered and excavated in 1899 and 1900) and completed (e.g. tail tip) with elements from a third partial skeleton (1903, Red Fork, ~100 miles north of Sheep Creek) [42]. The tail of *Diplodocus* consists of 70 caudal vertebrae, with a whip-like distal end making up 54.9% of the reconstructed total tail length. The terminal section lacks visible neural or transverse processes, indicating potential flexibility. Myhrvold & Currie [30] analysed the tails of various diplodocids and proposed that their exceptional length may have supported defensive behaviour or supersonic sound production for communication.

##### 2.2.2.3. *Varanus salvator*: Asian water monitor

The specimen of *V. salvator* from the Institute for Paleontology University Vienna (IPUW 3480) possesses a tail with 110 caudal vertebrae. The Asian water monitor’s tail is normally in contact with the ground and exhibits both lateral and dorsal movement through sinusoidal oscillation [43]. Typically, the tail measures 1.6 times the head–torso length, though this ratio varies depending on sex and population [43,44]. Similar to *Plateosaurus*, the tail of *V. salvator* progressively tapers into a whip-like end, which comprises 32.8% of the total tail length. It is well documented that monitor lizards use their tails primarily as a defensive weapon, with tail strikes capable of inflicting severe wounds on opponents [43]. However, given their role as generalist carnivores and scavengers, it is plausible that tail strikes may also serve offensive functions, for example during prey capture or aggressive interactions with conspecifics or competitors (compare [45]).

##### 2.2.2.4. *Iguana iguana*: green iguana

The tail of *I. iguana* accounts for approximately two-thirds of the total body length, though sexual dimorphism results in slight variation [46]. Similar to *V. salvator*, *I. iguana* is known to employ tail-whipping as a defensive behaviour [47]. The studied NHMW-ZOO-HS-1680 specimen of *I. iguana* possesses 39 caudal vertebrae, with the 39th forming a cone-like ossified structure. As in *Plateosaurus*, the spinal and transverse processes progressively diminish towards the distal tail, forming a whip-like tip. This whip-like end accounts for 37.1% of the total tail length, further supporting its role in rapid, flexible tail strikes for defence.

### 2.3. Measurement and energy calculations

For vertebral measurements, a caliper and measuring tape were used and photos were taken, which were additionally analysed with Fiji software [48]. All measurements are reported in centimetres (cm). Anatomical nomenclature and measurement protocols follow Huene [4], von den Driesch [49] and Moser [1].

To estimate the energy delivered by lateral tail impacts of *P. trossingensis*, a theoretical mathematical approach was applied [50,51]. The tails of *P. trossingensis* and the comparative taxa are modelled as elongated conical structures. In all species, a functional transition occurs where the caudal vertebrae become distinctly longer than they are tall, a morphological inflection interpreted here as the shift towards increased flexibility. We define this as the transition point (TP), a term used in this study to demarcate the beginning of the whip-like tail segment. While the specific concept of a TP is not established in prior literature, this definition is based on consistent vertebral morphology patterns in both fossil and extant reptiles, where elongated, gracile caudal vertebrae typically indicate enhanced lateral motion and tapering (cf. [43,52]). This anatomical shift justifies segmenting the tail into proximal and distal regions for biomechanical modelling. Measurements (electronic supplementary material, appendix S1) confirm that total vertebral height decreases caudally, while vertebral elongation increases proportionally with size reduction.

The kinetic energy (*E*), measured in joules (J), delivered by a whip-like tail strike is calculated using the equation: *E* = (1/2) × *m × v*^2^, where *m* represents the mass (grams, g) and *v* represents the velocity (metres per second, m s^−1^) [50,53]. The velocity estimates used in this study were based on general principles of dinosaur locomotion [54] and supported by comparative data from Conti *et al*. [55], who proposed tail-whip speeds of 1−2 m s^−1^ in large reptiles. To represent a high-impact scenario, the upper value of 2 m s^−1^ was selected. This value aligns with previous work by Gunga *et al*. [20], which estimated the average walking speed of *Plateosaurus* at approximately 3.7 km h^−1^ (~1 m s^−1^), suggesting that a 2 m s^−1^ tail-strike velocity is plausible for a rapid, localized movement of the distal tail.

Part of the total caudal vertebral lengths observed in *Plateosaurus* (and *Diplodocus*) in this study (see electronic supplementary material, appendix S1) shows irregularities that are likely attributable to a combination of taphonomic distortion and individual differences. Taphonomic processes such as compression and post-burial deformation can affect vertebral proportions, resulting in deviations from an expected progressive reduction in length. Identification of each vertebra’s position within the column was based on comparative morphology, articulation patterns and consistency in proportional relationships with adjacent vertebrae. For improved readability in comparative measurements, centimetres are converted to metres, grams to kilograms and joules to kilojoules in specific instances (see tables 1 and 2).

#### 2.3.1. Tail mass and volume estimation

To determine the total tail mass of *Plateosaurus*, including soft tissues, the general tail volume (Vol, in m³) is first estimated using the simplified cone-shape model: Vol = (1/3)𝜋𝑟^2^𝑙, where *r* (in cm) is half the height of the cranial-most caudal vertebra (Ca1) and *l* (in cm) is the total tail length [56,57]. The total tail length (*l*) was determined by manually measuring the length of each caudal vertebra up to the distal end. Missing vertebrae (Ca4, Ca9, Ca27 and Ca37) were estimated based on the mean length of adjacent vertebrae. Intervertebral discs and intervertebral space fillings were not included, following Mallison [9], who determined that no space allows for significant soft tissues between vertebral centres. To convert the simplified volume of the *Plateosaurus* tail into mass, a multiplication factor of 0.8 was applied. This is based on an assumption that 1000 cm³ has a specific weight of 0.8 kg, as established by Gunga *et al.* [20] in their total body mass reconstructions for *Plateosaurus* specimens.

#### 2.3.2. Energy calculation for defensive tail strikes

Multiple kinetic energy calculations were conducted to estimate the impact energy generated by defensive tail strikes in *Plateosaurus*, including (i) the total energy output of the whole tail when held horizontally, (ii) the energy transferred specifically from the whip-like tail segment and (iii) the energy delivered during a rapid, focused whiplash strike (see tables 1 and 2). Estimates that incorporate soft tissue were based on body composition models and density values proposed by Gunga *et al*. [20], who provided volumetric reconstructions and mass distribution data for *Plateosaurus*.

For soft tissue estimations, 35.9% muscle volume and 5.7% skin volume were added based on the reconstruction approach used by Gunga *et al.* [20]. To simplify calculations, the total volume (100%) was divided by the remaining 58.4%, yielding a scaling factor of ~1.7. This factor was applied to the cone radius in the volume equation to approximate soft tissue contributions, thereby refining the total kinetic energy transferred during a tail strike (figure 2).

#### 2.3.3. Comparative reference models

To contextualize the energy output of *Plateosaurus*, we calculated tail-strike energy for selected extinct and extant vertebrates using the same physical model 𝐸 = (1/2) × 𝑚 × *v*^2^. For *Diplodocus*, the bone-only tail mass (~33.2 kg) and strike velocity (104 m s^−1^) were derived from the biomechanical modelling of Myhrvold & Currie [30]. For extant reptiles, tail segment mass was approximated based on scaling relationships and allometric equations from body mass data in Campione & Evans [58]. Velocity estimates were informed by high-speed video analyses and behavioural studies: *V. salvator* shows tail-whip velocities of ~13–20 m s^−1^, while that of *I. iguana* typically reaches 5–10 m s^−1^ [25,43]. In all cases, the upper bounds of published velocity ranges were used to estimate maximum impact potential. The values used are summarized in table 2.

## 3. Results

### 3.1. Description of the preserved caudal vertebrae of the Vienna *Plateosaurus*

**Figure 3.**
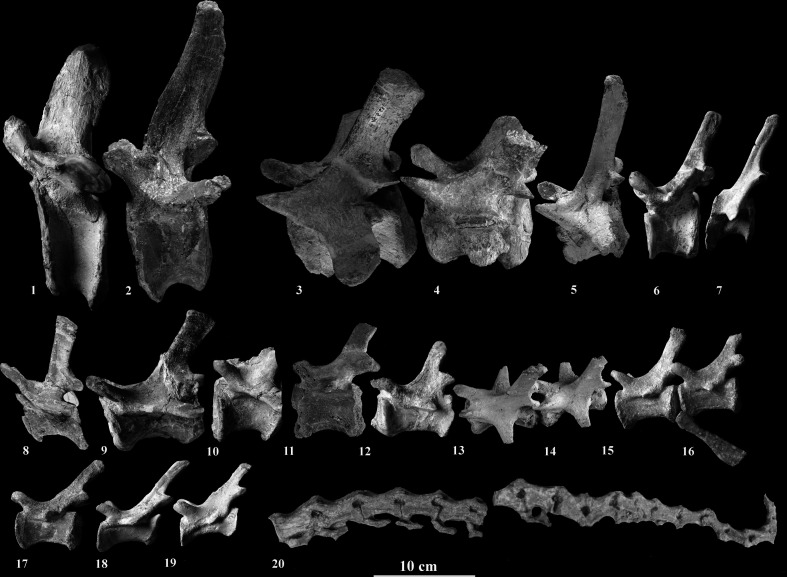
Preserved series of caudal vertebrae of the Vienna specimen of *Plateosaurus* with the field numbers from Frick: 1: 15.5.37, 2: 15.5.124, 3: 15.5.63 (mirrored), 4: 15.5.21 (mirrored), 5: 15.5.61, 6: 15.5.115, 7: 15.5.32, 8: 15.5.96, 9: 15.5.126, 10: 15.5.114, 11: 15.5.69, 12: 15.5.64, 13/14: 15.5.60 (mirrored), 15/16: 15.5.137, 17: 15.5.135,18: 15.5.62, 19: 15.5.136, 20: 15.5.22/24. All in lateral view.

The Vienna *Plateosaurus* specimen (Frick Field no. 15.5) preserves 43 caudal vertebrae, likely representing an almost complete tail (compared to specimens described by [4,9], which exhibit approximately 45 caudal vertebrae). However, several of the preserved vertebrae are deformed or incomplete due to diagenetic processes, and some are preserved only as corpora (figure 3).

The processus spinosus (neural spine) of the first two caudal vertebrae (Ca1, Ca2) is slightly inclined caudally and points dorsally. This caudal inclination increases progressively towards the middle of the tail (~Ca25) and is no longer present in the distal-most caudal vertebrae. Neural arches are preserved from Ca1 to Ca43, except where damaged by post-mortem processes. From Ca18 to the distal end, the vertebral corpora progressively flatten medio-laterally. The transverse processes vary in shape and orientation due to diagenetic deformation. In the first three caudal vertebrae, they remain robust and project horizontally. From approximately Ca25 onwards, the transverse processes are strongly reduced and take the form of low, elongated ridges along the lateral surfaces of the vertebrae. These ridges gradually decrease in size towards the tail tip, and their orientation appears to shift slightly caudally, giving the impression of being angled more towards the distal end rather than strictly horizontal. Additionally, the vertebral bodies decrease in size towards the mid-tail, reaching their maximum elongation around Ca26. From this point onwards, both size and length decrease gradually and evenly towards the distal end. Haemapophyses are present between the ventral articulation zones of individual caudal vertebrae, pointing distally and decreasing in size towards the terminal vertebrae. In total, 23 haemapophyses are preserved, though precise assignment is hindered by fractures and fragmentary preservation. Overall, the proximal tail of the Vienna specimen exhibits robust vertebral bodies and well-developed spinal processes

### 3.2. Calculation of the kinetic energy of the Vienna *Plateosaurus*

The whip-like tail of the Vienna *Plateosaurus* specimen accounts for 31.5% of the total estimated tail length and consists solely of caudal vertebrae with reduced processes (table 1). Following Mallison’s [9] calculations, sideways movements similar to a whip are biomechanically feasible. For estimating the kinetic energy output of a tail-lashing strike, the total tail length was calculated as 210 cm, with the whip-like portion measuring 62 cm, accounting for previously missing caudal vertebrae (Ca4, Ca9, Ca27 and Ca37). The estimated impact energy of the NHMW specimen of *P. trossingensis* was determined by applying physical parameters of velocity (*v*) and mass (*m*), following the kinetic energy equation described in §2 (table 1). The relative tail lengths and potential for tail-whipping were then compared across different taxa (table 2).

### 3.3. Calculation of the compared taxa

**Figure 4.**
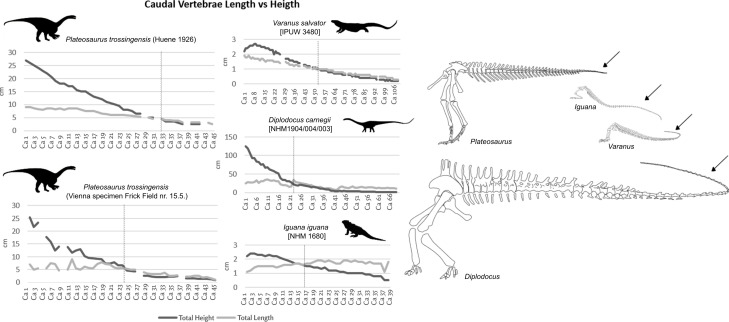
Caudal vertebrae length versus height of the Vienna specimen of *Plateosaurus trossingensis* (after [4]), *Plateosaurus trossingensis* Vienna specimen (Frick Field no. 15.5), *Varanus salvator* (IPUW 3480), *Diplodocus carnegii* (NHMW-Geo-1904/0004/0003, cast), *Iguana iguana* (NHMW-ZOO-HS-1680). All silhouettes at scale created by T.F. Arrows mark the whip tail.

All examined specimens and taxa exhibit variation in the total number of caudal vertebrae. Measurements indicate that, with the exception of *Diplodocus* (54.9%) and the *Plateosaurus* specimen described by Huene [4] (18.7%), the whip-tail proportion in the other taxa (*Plateosaurus* Vienna specimen, *V. salvator* and *I. iguana*) accounts for approximately 30–40% of the total tail length (figure 4; electronic supplementary material, appendix S1). These results suggest that the total number of caudal vertebrae does not significantly determine the relative length of the whip-tail. A notable discrepancy exists between the two *Plateosaurus* specimens, as the whip-tail accounts for ~40% of total tail length in Huene’s [4] specimen compared to 31.5% in the Vienna specimen. This variation may result from differences in the reconstructed positions of missing vertebrae. Huene [4] reported four missing caudal vertebrae between the 28th−29th, 30th−31st, 36th−37th and 39th−41st positions. The Vienna specimen also lacks four caudal vertebrae, but in different positions (Ca4, Ca9, Ca27 and Ca37). Additionally, potential variation in caudal vertebral morphology due to sex, ontogeny (age) or geographic distribution (Germany versus Switzerland) cannot be ruled out and may have influenced these observed differences.

The kinetic energy generated by the Vienna *Plateosaurus* specimen during a defensive tail strike was calculated using the formulas described in §2. The value of 56.14 kJ, listed in table 2 for *P. trossingensis* (Frick Field no. 15.5), reflects the estimated energy output of a full tail whip based on bony elements alone. When considering the caudal vertebrae of the entire tail including missing caudal vertebrae, the estimated kinetic energy reaches up to 60.207 kJ (see table 1). The estimate provides a theoretical range for the energy that could be transmitted through the tail during rapid lateral movement (table 1), assuming sufficient muscular input and tail flexibility. This would dramatically increase when adding soft tissue to the calculation; however, this is omitted in table 2 due to being hypothetical.

For all detailed measurements and comparative analysis of the studied tails, refer to tables 1 and 2 and electronic supplementary material, appendix S1.

## 4. Discussion

Tail-based defence in extinct reptiles is an underexplored topic, with most examples documented in a few fossil lineages such as ankylosaurs and glyptodonts [31,59,60]. In many taxa, tails serve multiple roles: locomotion, balance, and occasionally as a weapon. For *P. trossingensis*, which lacked obvious passive defences such as osteoderms or horn structures [4], an active defensive function of the tail warrants closer investigation.

This study shows that the impact energy of the whole tail of the Vienna *Plateosaurus* specimen when held horizontally could have generated a kinetic energy of 56.14 kJ, while the distal, whip-like portion of the tail alone could have generated significant kinetic energy during lateral strikes between 0.537 and 1.616 kJ (see table 1) depending on soft tissue estimates. As *Plateosaurus* was likely incapable of high-speed running, it is reasonable to assume that strike velocity would not vary substantially whether the animal was stationary or walking. These values are comparable to the energy outputs of defensive tail strikes in modern reptiles like *V. salvator* and *I. iguana* [25,43]. Modern reptiles display a wide range of stereotyped behaviours related to territory defence and predator deterrence, including tail lashing, body posturing and limb waving [61]. In both extant taxa, tail-whipping is used not only in defence but also in offensive contexts, including prey capture and intraspecific combat [17,62]. Given the generalist ecology of *Varanus*, it is plausible that tail strikes may serve more than one behavioural function. Applying this analogy cautiously to *Plateosaurus*, it is conceivable that tail-lashing was multifunctional used not only for deterring predators but also in aggressive interactions.

The lack of passive defences in *Plateosaurus* suggests it may have relied on a combination of behavioural strategies for survival. Its moderate body size (up to 10 m in length, ~1000 kg) placed it beyond the size range of most predators, but smaller individuals were likely vulnerable to contemporaneous carnivores such as *Liliensternus liliensterni* and *Notatesseraeraptor frickensis*. Tail-lashing could have played a role in mitigating predation risk, especially in juveniles or subadults. The total energy from a full tail sweep (up to ~174 kJ; see table 1) would have been sufficient to injure or deter small to mid-sized theropods. However, the absence of osteological trauma in predator fossils, or repetitive-use stress markers in *Plateosaurus* tails, limits the ability to confirm this behaviour.

A further speculative but intriguing possibility is that tail use could have extended to nest or juvenile defence. Parental care behaviours have been observed in extant reptiles and inferred in non-avian dinosaurs [63,64]. While there is no direct evidence for this in *Plateosaurus*, such scenarios remain biologically plausible and merit future investigation which should include ichnological data.

When compared to *Diplodocus*, whose elongated tail has been hypothesized to produce supersonic cracking [30], *Plateosaurus* shows a markedly different morphology. Its tail was shorter and more robust, favouring force over speed. This distinction likely reflects a different defensive function: while *Diplodocus* may have relied on high-velocity strikes for intimidation or communication, *Plateosaurus* would have employed slower but high-impact blows as a deterrent.

However, key limitations of this study must be acknowledged. The kinetic energy calculations are based on simplified biomechanical assumptions, using a conical tail model and estimated muscle volumes. They do not incorporate variables like tendon elasticity, energy transfer efficiency, or real-time strike dynamics. Furthermore, the behavioural inferences are necessarily indirect, drawn from anatomical modelling and comparisons with extant analogues.

**Figure 5.**
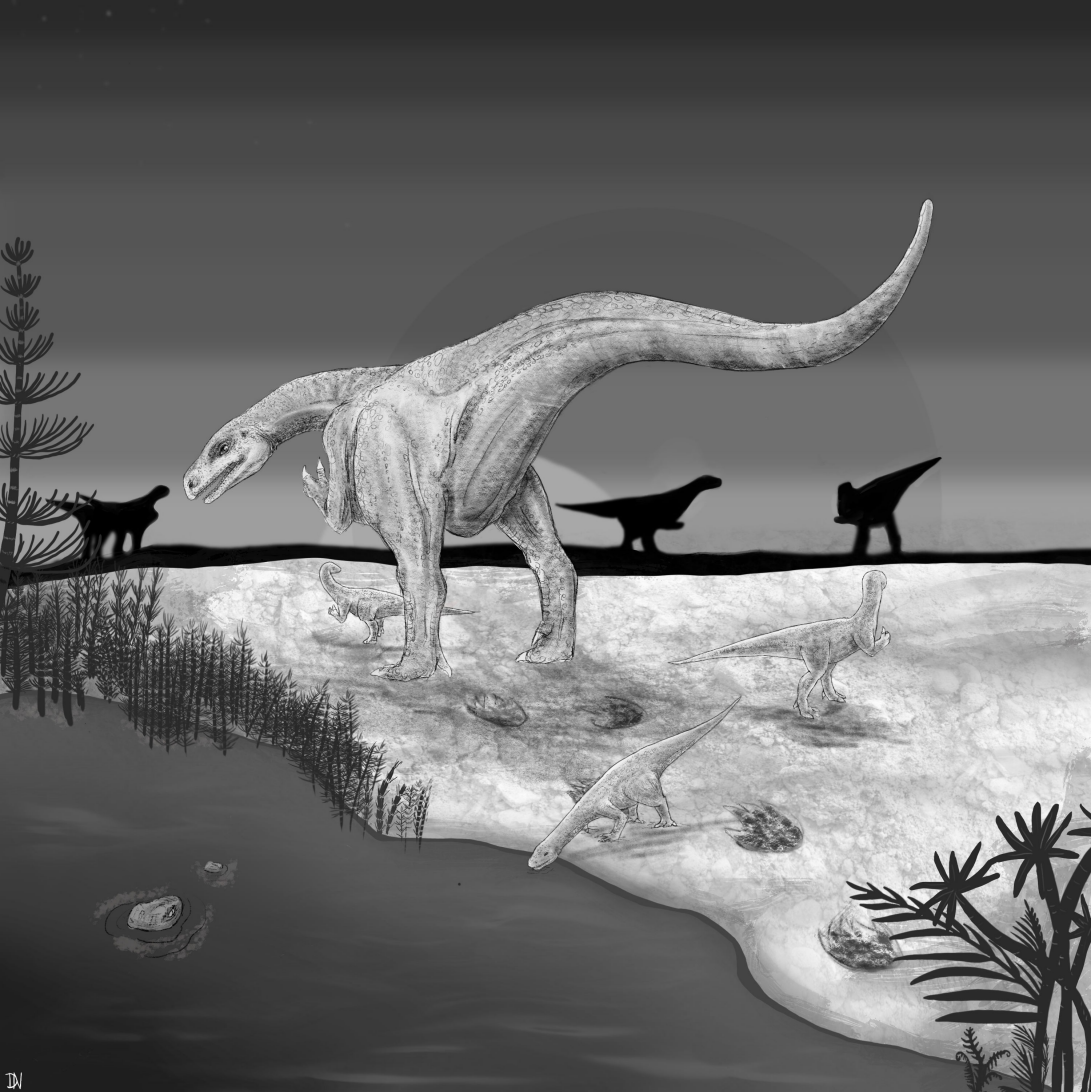
Reconstruction of a *Plateosaurus* herd featuring three juveniles and an adult, which is hypothetically depicted in the act of lashing its tail in defence against a predator (Copyright: Daria Filek and T.F.).

Finally, the recurring mass death assemblages of *Plateosaurus* at sites like Frick, Trossingen and Halberstadt have been interpreted both as evidence of gregarious behaviour and as taphonomic coincidences [3]. Even if group living existed, individual defensive behaviour such as tail-lashing would still have been advantageous. Tail strikes may have complemented other strategies such as body size, sociality or avoidance (figure 5).

## 5. Conclusion

This study offers new insights into the caudal morphology and potential defensive function of the tail in *Plateosaurus trossingensis*. Through vertebral measurements and biomechanical modelling, the plausibility of tail-lashing as a defensive behaviour was explored. While the findings suggest that the tail could have been used for active defence, this interpretation remains hypothetical in the absence of direct fossil evidence. By comparing *Plateosaurus* with both extant and extinct long-tailed taxa, the study contributes to a better understanding of tail function in early dinosaurs and highlights the importance of integrating anatomical and functional data in behavioural reconstructions.

## Data Availability

The paper presents new data in the form of bone measurements as electronic supplementary material, appendix S1 [65].
